# Association between self-reported eating speed and metabolic syndrome in a Beijing adult population: a cross-sectional study

**DOI:** 10.1186/s12889-018-5784-z

**Published:** 2018-07-11

**Authors:** Lixin Tao, Kun Yang, Fangfang Huang, Xiangtong Liu, Xia Li, Yanxia Luo, Lijuan Wu, Xiuhua Guo

**Affiliations:** 1 School of Public Health, Capital Medical University, Beijing, 100069 China; 2 Beijing Municipal Key Laboratory of Clinical Epidemiology, Beijing, 100069 China; 3 Department of Mathematics and Statistics, La Trobe University, Melbourne, 3086 Australia

**Keywords:** Metabolic syndrome, Eating speed, Elevated blood pressure, Central obesity, Dyslipidemia

## Abstract

**Background:**

Research on the relationship between metabolic syndrome (MetS), its components and eating speed is limited in China. The present study aimed to clarify the association between MetS, its components and eating speed in a Beijing adult population.

**Methods:**

This cross-sectional study included 7972 adults who were 18–65 years old and who received health check-ups at the Beijing Physical Examination Center in 2016. Logistic regression was conducted to explore the associations between MetS, its components and eating speed.

**Results:**

The prevalence of MetS in this population was 24.65% (36.02% for males and 10.18% for females). Eating speed was significantly associated with a high risk for MetS, elevated blood pressure, and central obesity for both genders. Eating speed was associated with a high risk for elevated triglycerides and with a reduction in high-density lipoprotein in males, and eating speed was associated with a high risk for elevated fasting plasma glucose in females. Compared with slow eating speed, the multivariate-adjusted odds ratios of medium eating speed and fast eating speed for MetS were 1.65 (95% confidence interval 1.32–2.07) and 2.27 (95% confidence interval 1.80–2.86) for all subjects, 1.58 (95% confidence interval 1.21–2.07) and 2.21 (95% confidence interval 1.69–2.91) for males, and 1.75 (95% confidence interval 1.15–2.68) and 2.27 (95% confidence interval 1.46–3.53) for females, respectively.

**Conclusions:**

Eating speed is positively associated with MetS and its components. Future recommendations aiming to prevent MetS and its components may focus on eating speed.

**Electronic supplementary material:**

The online version of this article (10.1186/s12889-018-5784-z) contains supplementary material, which is available to authorized users.

## Background

Metabolic syndrome (MetS) is a cluster of cardiometabolic abnormalities characterized by central obesity, elevated blood pressure (BP), elevated triglycerides (TG), elevated fasting plasma glucose (FPG) and reduced high-density lipoprotein (HDL) levels. MetS has now been recognized as a major health issue in most countries and across the world. Compared with the healthy population, people who have MetS are at approximately twice the risk for developing cardiovascular disease (CVD). Furthermore, MetS confers a 5-fold increase in the risk for type 2 diabetes mellitus [1, 2]. A meta-analysis reported that those with MetS have a higher risk for stroke [3]. Many clinical studies have also shown that MetS is significantly associated with a higher risk for all-cause mortality and for many other chronic diseases, such as mild cognitive impairment, coronary heart disease and nonalcoholic fatty liver disease [4–6].

MetS has reached epidemic proportions, and its prevalence is steadily increasing across the world. A previous study from the National Health and Nutrition Examination Survey reported that the prevalence of MetS, as assessed using the Joint Interim Statement criteria [1], increased from 25.3% in 1988–1994 to 34.2% in 2007–2012 in US adults [7]. MetS has now affected more than 20% of the adult population in China [8], Europe [9], and some developing countries [10, 11]. This medical condition has become a serious public health problem in China. A 1992 study reported that the prevalence of MetS was 13.3% in a population that included 27,739 subjects from 11 provinces of China [12]. The prevalence of MetS increased to 21.3% based on the 2009 China Health and Nutrition Survey [13].

Many previous studies have detected risk factors for MetS. These risk factors include socioeconomic factors, such as age, gender and income, and behavioural factors, such as nutrient intake, physical activity, smoking and drinking status [8, 10, 13]. Many other studies have also demonstrated that unhealthy eating habits, such as skipping breakfast, eating until full, preferring salty food, and smoking were risk factors for the development of MetS [14–16]. A number of studies have indicated that self-reported eating speed is not only positively associated with being overweight [17, 18] and with waist-to-height ratio [19] but also with insulin resistance [20], impaired glucose tolerance [21], type 2 diabetes [22], alanine amino-transferase activity [23, 24], nonalcoholic fatty liver disease [25, 26] and endoscopic erosive gastritis [27]. However, only a few studies have explored the association between eating speed and MetS [28–30]. Furthermore, most studies have focused on Japanese or Korean populations [28–32]. A population-based three-year cohort study showed that a fast eating speed is correlated with MetS, a large waist circumference (WC) and a low level of HDL [29]. A cross-sectional study of Korean men demonstrated that people with MetS are more likely to eat quickly than people without MetS [31]. Evidence from the Osaka Maternal and Child Health Study proved that a faster eating speed is positively associated with a high BMI in young Japanese children [32]. Another one-year, follow-up study among workers in the Tokyo Metropolitan area reported that if people change their eating speed from fast to slow, the risk of developing MetS will decrease [30].

No study has evaluated the association between eating speed and MetS in a Chinese adult population. Only one study has investigated the association between eating speed, energy intake and obesity in a population of Chinese preschool-aged children [33]. It is therefore necessary to examine the association between eating speed and MetS in a Chinese adult population. Therefore, the purpose of the present study was to clarify the association between MetS, its components and eating speed in an adult population in Beijing.

## Methods

### Study population

This cross-sectional study consisted of 16,580 participants who attended health check-ups at the Beijing Physical Examination Center in 2016. Among the 16,580 participants, we excluded subjects who did not complete the questionnaire (*n* = 8248), who were younger than 18 years old or older than 65 years old (*n* = 67), who had a previous diagnosis of CVD, who had cerebrovascular disease or cancer (*n* = 175) and who were missing information on their eating speed (*n* = 118). Finally, a total of 7972 subjects who were 18–65 years old, and who attended health check-ups at the Beijing Physical Examination Center were included in the study.

### Health examination and interview

The participants underwent physical examinations that included the measurement of height, weight, BP and overnight fasting blood sampling. Height was measured with a right-angle triangle resting on the scalp and against the wall to the nearest 0.5 cm, with the participant’s back square against the wall, with eyes looking straight ahead, and without shoes. Weight was measured without shoes and with a lever balance to the nearest 100 g. BP was measured for each subject in the sitting position after a 5-min rest period. During the 30 min preceding the measurements, the subjects were required to refrain from smoking and from ingesting caffeine. Three readings of systolic and diastolic BP were recorded with an interval of at least 1 min between readings, and the average of the last two measurements was used for data analysis. Several cuffs of different sizes were available to obtain accurate BP measurements in the hospital. A standard mercury sphygmomanometer was used in the hospital with 1 of 4 cuff sizes (paediatric, regular adult, large adult, or thigh) based on the participant’s arm circumference.

Blood samples were collected from subjects after an overnight fast of at least 12 h. HDL, TG and FPG were measured by an enzymatic method using a chemical analyser (Beckman LX 20, America) at the central laboratory of the hospital. All analyses were performed in accordance with the manufacturer’s recommendations.

Information about lifestyle factors, including education level, work stress, physical activity intensity, physical activity frequency, sleep duration, smoking status, drinking status, excessive salt intake, excessive sugar intake, excessive fat intake, excessive meat intake, a mainly vegetable diet, frequency of eating breakfast, grain consumption, and a history of antihypertensive, antidiabetic and hypolipidaemic treatment were collected using a self-administered questionnaire. Information concerning eating speed was based on three possible responses (slow, medium, fast) to the question “How fast is your eating speed compared to others?”

### Definition of MetS

MetS was diagnosed if the subjects had three or more risk factors according to the Joint Interim Statement criteria [1]. The risk factors were as follows:

1. Central obesity: WC ≥85 cm for males and WC ≥ 80 cm for females;
2. Elevated TG (drug treatment for elevated TG is an alternative indicator) ≥ 150 mg/dL (1.7 mmol/L);
3. Reduced HDL (drug treatment for reduced HDL is an alternative indicator) < 40 mg/dL (1.0 mmol/L) for males and < 50 mg/dL (1.3 mmol/L) for females;
4. Elevated BP (antihypertensive drug treatment in a patient with a history of hypertension is an alternative indicator), systolic BP ≥ 130 mmHg and/or diastolic BP ≥ 85 mmHg; and
5. Elevated FPG (drug treatment of elevated glucose is an alternative indicator) ≥ 100 mg/dL.

### Missing data

The problem of missing data often occurs in observational studies, possibly leading to biased results. Therefore, differences in variables between data with and without completing a questionnaire were statistically tested and were reported.

### Statistical analyses

Data are presented as the median (25, 75th percentile) for continuous variables or the number (%) for categorical variables. The characteristics of the participants were compared among three eating speed groups. Cochran-Armitage trend tests were used to detect whether or not there was a linear correlation between the prevalence of MetS and its components with the increasing eating speed. Kruskal–Wallis rank tests were used for non-normally distributed continuous variables in the three groups. Chi-square tests were used for nominal categorical variables, and Kruskal–Wallis rank tests were used for ordinal categorical variables in the three groups. Logistic regression analyses were performed to estimate the odds ratios (ORs) and 95% confidence intervals (CIs) of the risk for MetS and its components based on eating speed.

We fitted the multivariate models with adjustments for the following potential confounders: age (years), education level (high school or lower, college, graduate education or above), work stress (low, moderate, high), physical activity intensity (sedentary, low, moderate, high), physical activity frequency (sedentary, less than once per week, more than once per week, more than once every day), sleep duration (< 6 h, 6~ 8 h, > 8 h), smoking status (current or former, never), drinking status (current or former, never), excessive salt intake (yes, no), excessive sugar intake (yes, no), excessive fat intake (yes, no), excessive meat intake (yes, no), a mainly vegetable diet (yes, no), frequency of eating breakfast (skip breakfast, less than three times per week, about five times per week, almost every day), grain consumption (mainly coarse grain, mainly refined grain, half and half), a history of antihypertensive treatment (yes, no), a history of antidiabetic treatment (yes, no) and a history of hypolipidaemic treatment (yes, no). The first category for these categorical variables was considered the reference category. We also fitted the multivariate models with the second category of eating speed as the reference category.

All statistical analyses were performed using SAS (Version 9.2; SAS Institute, Chicago, IL, USA), and *P* < 0.05 was considered statistically significant.

## Results

### Prevalence of MetS and its components

**Fig. 1:**
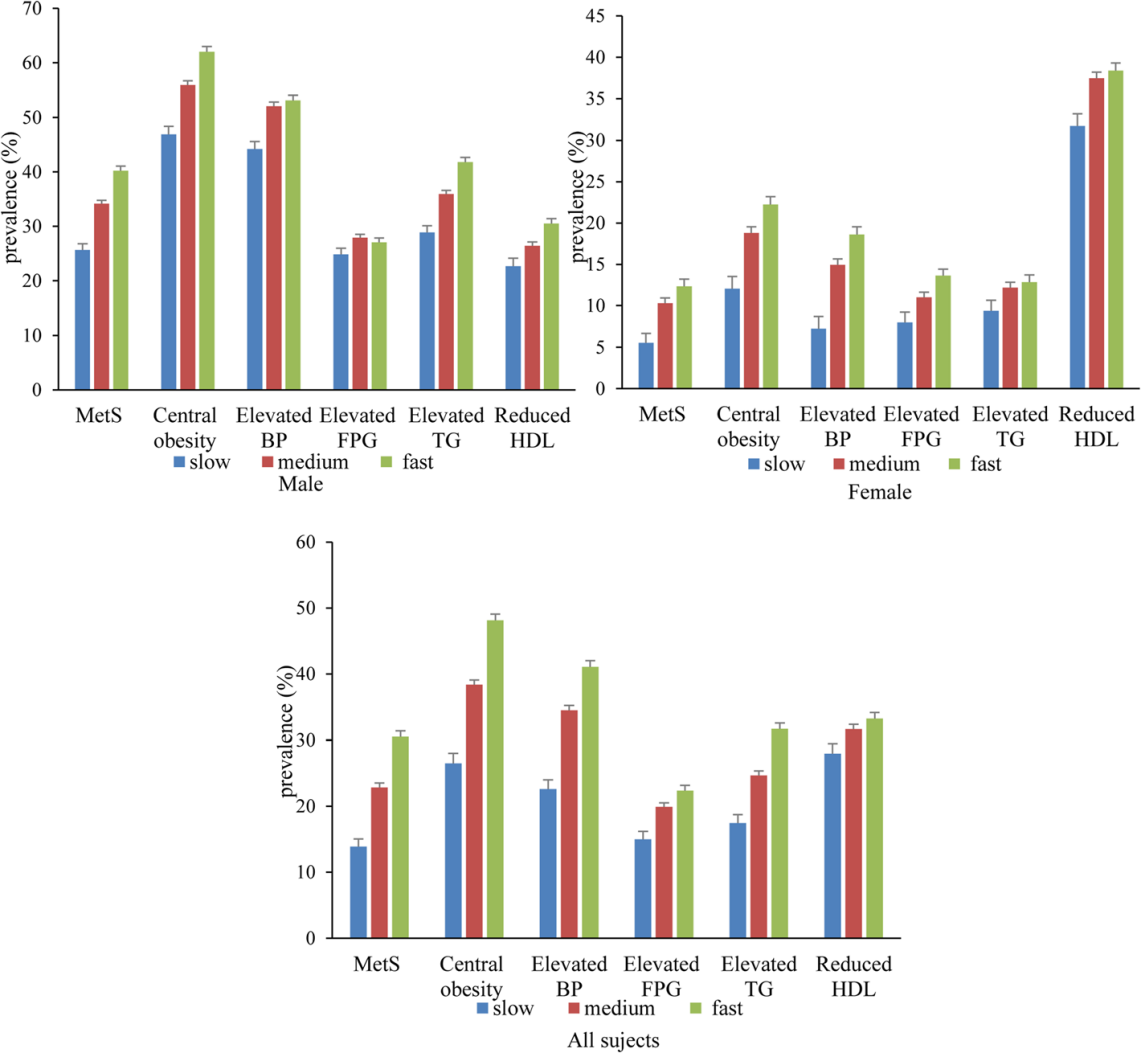
The prevalence of MetS and its components by eating speed. MetS: metabolic syndrome; BP: blood pressure; FPG: fasting plasma glucose; TG: triglycerides; HDL: high-density lipoprotein

The prevalence of MetS and its components are shown in Fig. 1. Of the 7972 subjects, 1965 (24.65%) subjects met the criteria for MetS. For the MetS components, 3237 (40.60%) subjects met the criteria for central obesity, 2682 (33.64%) subjects met the criteria for elevated BP, 1582 (19.84%) subjects met the criteria for elevated FPG, 2011 (25.23%) subjects met the criteria for elevated TG, and 2427 (30.44%) subjects met the criteria for reduced HDL. Except for the prevalence of elevated FPG for males and elevated TG for females, the prevalence of MetS and its other components increased as eating speed increased for both genders (*P* for trend < 0.05).

### Characteristics of study participants

The general characteristics of the study participants can be found in Tables 1, 2 and 3. Participants who ate faster were more likely to be younger males, and to have a higher BMI and WC. Participants who ate faster were also more likely to have a higher TG level, SBP, and DBP and a lower HDL level. History of antidiabetic, hypolipidaemic treatment, physical activity frequency, smoking status, excessive sugar intake, a mainly vegetable diet, and frequency of eating breakfast did not significantly differ with eating speed for both genders. However, there were significant differences in work stress, excessive salt intake and grain consumption among the three eating speed groups for both genders. History of antihypertensive treatment, physical activity intensity and drinking status did not significantly differ among the three eating speed groups for males. Education level, sleep duration, excessive fat intake and excessive meat intake did not significantly differ among the three eating speed groups for females.

**Table 1:** Characteristics of study participants by eating speed

Variable	Male				Female			
	Slow (*n* = 371)	Medium (*n* = 2188)	Fast (*n* = 1905)	*P* value^a^	Slow (*n* = 523)	Medium (*n* = 1964)	Fast (*n* = 1021)	*P* value^a^
Age (years)^b^	38 (31–48)	39 (32–48)	37 (31–46)	< 0.0001	34 (29–41)	35 (30–43)	36 (30–43)	0.0041
BMI (kg/m^2^)^b^	24.2 (21.8–26.8)	25.0 (23.1–27.0)	25.7 (23.5–28.1)	< 0.0001	21.1 (19.1–23.1)	21.8 (20.2–24.1)	22.5 (20.5–24.8)	< 0.0001
WC (cm)^b^	84 (78–92)	87 (81–93)	88 (83–95)	< 0.0001	70 (65–76)	72 (68–78)	73 (68–79)	< 0.0001
SBP (mm Hg)^b^	120 (110–130)	120 (116–130)	120 (110–130)	0.0009	110 (100–120)	110 (100–120)	110 (100–120)	< 0.0001
DBP (mm Hg)^b^	80 (80–90)	80 (80–90)	84 (80–90)	0.0049	70 (70–80)	80 (70–80)	80 (70–80)	0.0001
FPG (mmol/L)^b^	5.22 (4.94–5.58)	5.23 (4.92–5.64)	5.23 (4.93–5.63)	0.7603	4.95 (4.70–5.25)	5.02 (4.74–5.33)	5.01 (4.74–5.33)	0.0162
TG (mmol/L)^b^	1.18 (0.81–1.81)	1.34 (0.89–1.98)	1.47 (0.98–2.26)	< 0.0001	0.79 (0.60–1.14)	0.86 (0.62–1.23)	0.84 (0.63–1.23)	0.0300
HDL (mmol/L)^b^	1.19 (1.03–1.39)	1.15 (1.01–1.31)	1.11 (0.98–1.27)	< 0.0001	1.45 (1.27–1.62)	1.40 (1.22–1.61)	1.39 (1.22–1.57)	0.0065
Education (*n*, %)				0.0021				0.1374
High school or lower	73 (19.68)	371 (16.96)	250 (13.12)		32 (6.12)	151 (7.69)	57 (5.58)	
College	235 (63.34)	1431 (65.40)	1294 (67.93)		343 (65.58)	1209 (61.56)	647 (63.37)	
Graduate or above	63 (16.98)	386 (17.64)	361 (18.95)		148 (28.30)	604 (30.75)	317 (31.05)	
Work stress (*n*, %)				< 0.0001				0.0001
Low	69 (18.60)	286 (13.07)	244 (12.81)		90 (17.21)	310 (15.78)	133 (13.03)	
Moderate	142 (38.38)	1001 (45.94)	706 (37.32)		267 (51.35)	955 (48.80)	463 (45.66)	
High	160 (43.13)	901 (41.18)	955 (50.13)		166 (31.74)	699 (35.59)	425 (41.63)	
History of antihypertensive treatment (*n*, %)	29 (7.82)	235 (10.74)	209 (10.97)	0.1867	7 (1.34)	65 (3.31)	43 (4.21)	0.0110
History of antidiabetic treatment (*n*, %)	8 (2.16)	87 (3.98)	69 (3.62)	0.2238	2 (0.38)	16 (0.81)	15 (1.47)	0.0764
History of hypolipidaemic treatment (*n*, %)	7 (1.89)	54 (2.47)	54 (2.83)	0.5190	4 (0.76)	30 (1.53)	18 (1.76)	0.2979

*BMI* body mass index, *WC* waist circumference, *SBP* systolic blood pressure, *DBP* diastolic blood pressure, *FPG* fasting plasma glucose, *TG* triglycerides, *HDL* high-density lipoprotein

^a^Chi-square test or Kruskal-Wallis rank test

^b^Values are expressed as the median (P_25_-P_75_)

**Table 2:** Behavioural factors of study participants by eating speed

Variable	Male				Female			
	Slow (*n* = 371)	Medium (*n* = 2188)	Fast (*n* = 1905)	*P* value^a^	Slow (*n* = 523)	Medium (*n* = 1964)	Fast (*n* = 1021)	*P* value^a^
Physical activity intensity (*n*, %)				0.4913				0.0454
Sedentary	69 (18.65)	353 (16.22)	354 (18.72)		122 (23.42)	444 (22.68)	273 (26.82)	
Low	144 (38.92)	960 (44.12)	810 (42.83)		278 (53.36)	1057 (53.98)	532 (52.26)	
Moderate	120 (32.43)	610 (28.03)	489 (25.86)		96 (18.43)	367 (18.74)	168 (16.50)	
High	37 (10.00)	253 (11.63)	238 (12.59)		25 (4.80)	90 (4.60)	45 (4.42)	
Physical activity frequency (*n*, %)				0.3841				0.5884
Sedentary	51 (13.78)	211 (9.69)	235 (12.39)		77 (14.75)	277 (14.14)	153 (15.06)	
Less than once per week	129 (34.86)	806 (37.02)	676 (35.64)		215 (41.19)	749 (38.23)	393 (38.68)	
More than once per week	104 (28.11)	715 (32.84)	605 (31.89)		148 (28.35)	637 (32.52)	315 (31.00)	
More than once per day	86 (23.24)	445 (20.44)	381 (20.08)		82 (15.71)	296 (15.11)	155 (15.26)	
Sleep duration (*n*, %)				0.0011				0.2983
< 6 h	336 (90.57)	1959 (89.53)	1639 (86.04)		479 (91.59)	1795 (91.40)	916 (89.72)	
6 ~ 8 h	22 (5.93)	1959 (89.53)	227 (11.92)		25 (4.78)	121 (6.16)	79 (7.74)	
> 8 h	13 (3.50)	36 (1.65)	39 (2.05)		19 (3.63)	48 (2.44)	26 (2.55)	
Smoking status (*n*, %)	143 (38.54)	932 (42.60)	779 (40.89)	0.2586	12 (2.29)	32 (1.63)	24 (2.35)	0.3249
Drinking status (*n*, %)	225 (60.65)	1385 (63.30)	1184 (62.15)	0.5420	41 (7.84)	228 (11.61)	152 (14.89)	0.0002

^a^: Chi-square test or Kruskal-Wallis rank test

**Table 3:** Dietary factors of study participants by eating speed

Variables	Male				Female			
	Slow (*n* = 371)	Medium (*n* = 2188)	Fast (*n* = 1905)	*P* value^a^	Slow (*n* = 523)	Medium (*n* = 1964)	Fast (*n* = 1021)	*P* value^a^
Excessive salt intake (*n*, %)	104 (28.03)	494 (22.58)	560 (29.40)	< 0.0001	63 (12.05)	293 (14.92)	221 (21.65)	< 0.0001
Excessive sugar intake (*n*, %)	28 (7.55)	154 (7.04)	169 (8.87)	0.0917	58 (11.09)	200 (10.18)	113 (11.07)	0.6952
Excessive fat intake (*n*, %)	19 (5.12)	103 (4.71)	138 (7.24)	0.0021	19 (3.63)	59 (3.00)	47 (4.60)	0.0817
Excessive meat intake (*n*, %)	66 (17.79)	365 (16.68)	414 (21.73)	0.0002	46 (8.80)	161 (8.20)	102 (9.99)	0.2608
A mainly vegetable diet (*n*, %)	54 (14.56)	257 (11.75)	201 (10.55)	0.0733	90 (17.21)	335 (17.06)	170 (16.65)	0.9486
Frequency of eating breakfast (*n*, %)				0.3533				0.1096
Skip breakfast	16 (4.34)	89 (4.10)	75 (3.96)		11 (2.12)	26 (1.33)	20 (1.97)	
Less than three times per week	36 (9.76)	218 (10.05)	195 (10.30)		18 (3.46)	77 (3.95)	43 (4.24)	
About five times per week	46 (12.47)	345 (15.90)	262 (13.84)		44 (8.46)	217 (11.12)	123 (12.12)	
Almost every day	271 (73.44)	1518 (69.95)	1361 (71.90)		447 (85.96)	1631 (83.60)	829 (81.67)	
Grain consumption (*n*, %)				< 0.0001				0.0130
Mainly coarse grain	56 (15.22)	215 (9.90)	92 (4.87)		62 (11.90)	179 (9.16)	79 (7.77)	
Mainly refined grain	196 (53.26)	1319 (60.76)	1340 (70.86)		290 (55.66)	1106 (56.60)	627 (61.65)	
Half and half	116 (31.52)	637 (29.34)	459 (24.27)		169 (32.44)	669 (34.24)	311 (30.58)	

^a^:Chi-square test or Kruskal-Wallis rank test

### Association between eating speed and MetS

The association between eating speed and MetS was explored after adjusting for age, education level, work stress, physical activity intensity, physical activity frequency, sleep duration, smoking status, drinking status, excessive salt intake, excessive sugar intake, excessive fat intake, excessive meat intake, a mainly vegetable diet, frequency of eating breakfast, grain consumption, and a history of antihypertensive, antidiabetic, and hypolipidaemic treatment (Table 4).

**Table 4:** The adjusted association between MetS, its components and eating speed

Gender	Variables	Eating speed			*P* for trend
		Slow	Medium	Fast	
All	MetS	Reference	1.65 (1.32–2.07)	2.27 (1.80–2.86)	< 0.0001
	Central obesity	Reference	1.39 (1.12–1.72)	1.68 (1.35–2.09)	< 0.0001
	Elevated BP	Reference	1.57 (1.28–1.92)	1.82 (1.48–2.24)	< 0.0001
	Elevated FPG	Reference	1.11 (0.88–1.41)	1.18 (0.93–1.51)	0.1644
	Elevated TG	Reference	1.25 (1.00–1.55)	1.51 (1.21–1.88)	< 0.0001
	Reduced HDL	Reference	1.22 (1.02–1.45)	1.33 (1.11–1.60)	0.0026
Male	MetS	Reference	1.58 (1.21–2.07)	2.21 (1.69–2.91)	< 0.0001
	Central obesity	Reference	1.27 (0.97–1.67)	1.53 (1.16–2.02)	0.0008
	Elevated BP	Reference	1.37 (1.08–1.75)	1.50 (1.17–1.92)	0.0037
	Elevated FPG	Reference	1.13 (0.85–1.50)	1.14 (0.85–1.52)	0.5278
	Elevated TG	Reference	1.30 (0.99–1.71)	1.69 (1.29–2.23)	< 0.0001
	Reduced HDL	Reference	1.11 (0.84–1.47)	1.35 (1.02–1.79)	0.0038
Female	MetS	Reference	1.75 (1.15–2.68)	2.27 (1.46–3.53)	0.0002
	Central obesity	Reference	1.60 (1.12–2.28)	1.98 (1.36–2.88)	0.0004
	Elevated BP	Reference	2.11 (1.42–3.13)	3.02 (2.00–4.56)	< 0.0001
	Elevated FPG	Reference	1.15 (0.78–1.68)	1.56 (1.05–2.33)	0.0079
	Elevated TG	Reference	1.14 (0.79–1.65)	0.95 (0.63–1.43)	0.5326
	Reduced HDL	Reference	1.28 (0.82–1.61)	1.20 (0.94–1.54)	0.3288

All models were adjusted for age, education level, work stress, physical activity intensity, physical activity frequency, sleep duration, smoking status, drinking status, excessive salt intake, excessive sugar intake, excessive fat intake, excessive meat intake, a mainly vegetable diet, frequency of eating breakfast, grain consumption, and a history of antihypertensive, antidiabetic, and hypolipidaemic treatment

*MetS* metabolic syndrome, *BP* blood pressure, *FPG* fasting plasma glucose, *TG* triglycerides, *HDL* high-density lipoprotein

Eating speed was statistically associated with the prevalence of MetS for all study subjects. Compared with slow eating speed, ORs and 95% CIs for medium eating speed and fast eating speed were 1.65 (1.32–2.07) and 2.27 (1.80–2.86), respectively, for all subjects (*p* for trend < 0.001). For both males and females, eating speed was statistically associated with the risk for developing MetS. Compared with slow eating speed, the ORs and 95% CIs for medium eating speed and fast eating speed were 1.58 (1.21–2.07) and 2.21 (1.69–2.91), respectively, for males (*p* for trend < 0.001). Compared with slow eating speed, the ORs and 95% CIs for medium eating speed and fast eating speed were 1.75 (1.15–2.68) and 2.27 (1.46–3.53), respectively, for females (*p* for trend < 0.001).

### Association between eating speed and MetS components

The associations between eating speed and MetS components were also investigated after adjusting for confounding factors (Table 4). Eating speed was significantly associated with central obesity and elevated BP for all subjects and both genders. However, no association between eating speed and elevated FPG was found for the whole population and males in the present study. Furthermore, no association was revealed between elevated TG, reduced HDL and eating speed for females in this study.

### Missing data

Differences in variables between data with and without completing the questionnaire were statistically tested and reported in Additional file 1: Table S1. Subjects who did not complete a questionnaire were significantly more likely to be older and were more likely to have elevated FPG and TG. No statistical difference was found in the gender ratio, the prevalence of MetS, central obesity, elevated BP and reduced HDL between subjects who did or did not complete a questionnaire.

### Sensitivity analysis

We also conducted a multivariate logistic regression to explore the association between eating speed and MetS, as well as its component, with medium eating speed as the reference level (Additional file 2: Table S2). It was shown that there were statistically significant differences between the medium and fast eating speed groups in the associations between MetS, central obesity, elevated BP, elevated TG and eating speed for all subjects. For males, there were statistically significant differences between the medium and fast eating speed groups in the associations between MetS, central obesity, elevated TG, reduced HDL and eating speed. For females, there were statistically significant differences between the medium and fast eating speed groups in the associations between MetS, elevated BP, elevated FPG and eating speed.

For Mets and obesity, defined as a BMI equal to or above 30 kg/m^2^, multivariate logistic regression models were also used to assess the association between MetS, central obesity and eating speed (Additional file 3: Table S3). The results were relatively consistent with that of using WC as the criteria.

## Discussion

To our knowledge, this is the first study to investigate the association between MetS, its components and eating speed in a Chinese adult population. The results showed that eating speed might increase the risk for MetS, central obesity and elevated BP for both genders. However, the present study demonstrated that eating speed may increase the risk for elevated TG and reduced HDL for males only and may increase the risk for elevated FPG for females only.

In this present study, the prevalence of MetS was similar to that found in a previous study in China [12]. Central obesity was the most prevalent among the five MetS components at 40.60% in this study, which was also found in the 2011 China Health and Nutrition Survey study [34]. The prevalence of elevated BP, elevated FPG, elevated TG and reduced HDL were relatively consistent with those in previous research as well [12].

Our results showed that eating speed is associated with a high risk for MetS after adjusting for confounding factors. The same result was reported in a Japanese study [29]. The Japanese study reported that a fast eating speed is associated with a high risk for the incidence of MetS after adjustment for potential confounding factors. Among the metabolic components, eating speed was statistically associated with a high WC and reduced HDL. Our results also indicated that eating fast is closely associated with obesity for all subjects, both males and females. Numerous previous studies also reported similar results [17–19]. The present study found that eating fast is associated with elevated BP, which is consistent with a previous study [35]. Our study demonstrated the association between eating speed and elevated FPG for females, which was also suggested in previous studies [21, 35]. One study that focused on middle-aged Japanese men reported adjusted hazard ratios of 1.68 and 1.97 for medium and fast eating speed, respectively, and it reported that eating speed is associated with the risk for type 2 diabetes [36]. Another study showed that those who ate fast were at a higher risk for the incidence of impaired glucose tolerance than those who ate slowly [21]. These results suggest that a self-reported fast eating speed is a potential risk factor for developing impaired glucose tolerance. In addition, our study showed that eating fast was associated with elevated TG and reduced HDL for all subjects and males. No statistically significant association was found between eating speed and elevated TG or reduced HDL for females. Previous studies also reported the association between lipid abnormality and eating speed [28, 29]. However, the gender-specific association between elevated FPG, lipid abnormality and eating speed needs to be verified in large cohort studies.

Several mechanisms can explain why eating fast is associated with a high risk for MetS and its components. Laboratory studies have shown that a fast eating speed can increase the level of serum insulin resistance [20], leading to MetS and high levels of FPG. Eating fast can lead to overeating, resulting in excess food intake, higher postprandial blood glucose [20], overweight [17–19] and MetS. The HDL level can be affected by fast eating speed via a greater energy intake. If people eat fast, calories pass through the oral cavity quickly and do not initiate a feeling of fullness, resulting in an increased calorie intake [37]. A previous study reported that a low calorie diet can increase the HDL level and improve other lipid profiles [38]. One study showed that interleukin-1β and interleukin-6 levels were higher among those who ate quickly than among those who ate slowly, even after accounting for energy intake and BMI [39]. These cytokines could induce insulin resistance [20, 40], contributing to high blood pressure via increased renal sodium and water retention, plasma noradrenaline and sympathetic nervous system activity [41].

The strength of this study is that it is the first study to explore the association between MetS, its components and eating speed in a Chinese adult population. The present study investigated the association between MetS, its components and eating speed after adjusting for many confounding factors, including age, education level, occupation, work stress, physical activity intensity, physical activity frequency, sleep duration, smoking status, drinking status, excessive salt intake, excessive sugar intake, excessive fat intake, excessive meat intake, a mainly vegetable diet, frequency of eating breakfast, grain consumption, and a history of antihypertensive, antidiabetic, and hypolipidaemic treatment. Thus, the results are more credible. Finally, the study evaluated the associations between each component of MetS and eating speed, generating more specific information for future intervention studies.

There are three limitations in the present study. The study was cross-sectional, and a longitudinal study may be more meaningful in exploring the causal association between MetS, its components and eating speed. The second limitation was that energy intake was not adjusted for in the present study. Future studies should focus on how eating speed affects MetS after adjusting for total energy intake. The third limitation of the present study was missing data. Subjects who did not complete a questionnaire were excluded from the study, and it may have biased the results. In future studies, we will take measures to increase the response rate of completing the questionnaire.

## Conclusions

Eating speed is associated with a high risk for MetS, elevated BP, and central obesity for both genders. However, eating speed is associated with a high risk for elevated TG and reduced HDL in males only; eating speed is associated with a high risk for elevated FPG in females only. Future recommendations to prevent MetS and its components should focus on eating speed.

## Additional files

Additional file 1: **Table S1.** Variable differences between data with and without questionnaire. (DOCX 16 kb)

Additional file 2: **Table S2.** The adjusted association between MetS, its components and eating speed using medium eating speed as the reference level. (DOCX 20 kb)

Additional file 3: **Table S3.** The adjusted association between MetS, obesity and eating speed. (DOCX 14 kb)

## Abbreviations

BMI: Body mass index
BP: Blood pressure
CI: Confidence interval
CVD: Cardiovascular disease
DBP: Diastolic blood pressure
FPG: Fasting plasma glucose
HDL: High-density lipoproteins
MetS: Metabolic syndrome
OR: Odds ratio
SBP: Systolic blood pressure
TG: Triglycerides
WC: Waist circumference
