# Supplementary material for: Association between self-reported eating speed and metabolic syndrome in a Beijing adult population: a cross-sectional study
Source: BMC Public Health. 2018 Jul 11;18:855. doi: 10.1186/s12889-018-5784-z (PMC6042428; doi:10.1186/s12889-018-5784-z)
Supplement: Supplementary file 1 — Table S1. Variable differences between data with and without questionnaire. (DOCX 16 kb) [file 12889_2018_5784_MOESM1_ESM.docx]

**Table S1** Variable differences between data with and without questionnaire

| Variables | With completing questionnaire  (*n*=7972) | Without completing questionnaire  (*n*=8248) | *P* value |
| --- | --- | --- | --- |
| Male (*n*, %) | 4464 (56.00) | 4701 (57.00) | 0.1992*^a^* |
| Age (years) | 37 (31-46) | 39 (31-51) | <0.000*^b^* |
| BMI (kg/m^2^) | 23.90 (21.40-26.40) | 23.90 (21.60-26.40) | 0.3574*^b^* |
| WC (cm) | 81 (72-90) | 81 (73-89) | 0.4867*^b^* |
| SBP (mm Hg) | 120 (110-130) | 120 (110-130) | 0.3622 *^b^* |
| DBP (mm Hg) | 80 (70-86) | 80 (70-86) | 0.2037*^b^* |
| FPG (mmol/L) | 5.13 (4.83-5.49) | 5.19 (4.89-5.55) | <0.0001*^b^* |
| TG (mmol/L) | 1.09 (0.74-1.70) | 1.15 (0.77-1.77) | <0.0001*^b^* |
| HDL (mmol/L) | 1.24 (1.07-1.46) | 1.25 (1.07-1.47) | 0.1902*^b^* |
| MetS (*n*, %) | 1965 (24.65) | 1958 (23.74) | 0.1762*^a^* |
| Central obesity (*n*, %) | 3237 (40.60) | 3384 (41.03) | 0.5833*^a^* |
| Elevated BP (*n*, %) | 2682 (33.64) | 2815 (34.13) | 0.5126*^a^* |
| Elevated FPG (*n*, %) | 1582 (19.84) | 1796 (21.77) | 0.0025*^a^* |
| Elevated TG (*n*, %) | 2011 (25.23) | 2325 (28.19) | <0.0001*^a^* |
| Reduced HDL (*n*, %) | 2427 (30.44) | 2510 (30.43) | 0.9863*^a^* |

*^a^*:Chi-square test *^b^*:Wilcoxon rank test

BMI: body mass index; WC: waist circumference; SBP: systolic blood pressure; DBP: diastolic blood pressure; FPG: fasting plasma glucose; TG: triglycerides; HDL: high-density lipoprotein; BP: blood pressure
