# Supplementary material for: Association between self-reported eating speed and metabolic syndrome in a Beijing adult population: a cross-sectional study
Source: BMC Public Health. 2018 Jul 11;18:855. doi: 10.1186/s12889-018-5784-z (PMC6042428; doi:10.1186/s12889-018-5784-z)
Supplement: Supplementary file 2 — Table S2. The adjusted association between MetS, its components and eating speed using medium eating speed as the reference level. (DOCX 20 kb) [file 12889_2018_5784_MOESM2_ESM.docx]

**Table S2** The adjusted association between MetS, its components and eating speed using medium eating speed as the reference level

| Gender | Variables | Eating speed | | |
| --- | --- | --- | --- | --- |
|  |  | Slow | Medium | Fast |
| All | MetS | 0.60 (0.48-0.76) | Reference | 1.37 (1.22-1.55) |
|  | Central obesity | 0.72 (0.58-0.89) | Reference | 1.21 (1.06-1.37) |
|  | Elevated BP | 0.64 (0.52-0.78) | Reference | 1.16 (1.03-1.31) |
|  | Elevated FPG | 0.90 (0.71-1.14) | Reference | 1.06 (0.93-1.22) |
|  | Elevated TG | 0.80 (0.65-1.00) | Reference | 1.21 (1.07-1.37) |
|  | Reduced HDL | 0.82 (0.69-0.98) | Reference | 1.10 (0.98-1.22) |
| Male | MetS | 0.63 (0.48-0.83) | Reference | 1.40 (1.22-1.61) |
|  | Central obesity | 0.79 (0.60-1.03) | Reference | 1.21 (1.03-1.41) |
|  | Elevated BP | 0.73 (0.57-0.93) | Reference | 1.09 (0.95-1.25) |
|  | Elevated FPG | 0.88 (0.65-1.18) | Reference | 0.99 (0.84-1.17) |
|  | Elevated TG | 0.78 (0.59-1.02) | Reference | 1.31 (1.14-1.52) |
|  | Reduced HDL | 0.90 (0.68-1.19) | Reference | 1.22 (1.05-1.41) |
| Female | MetS | 0.57 (0.37-0.87) | Reference | 1.29 (1.00-1.67) |
|  | Central obesity | 0.63 (0.44-0.89) | Reference | 1.24 (0.99-1.56) |
|  | Elevated BP | 0.47 (0.32-0.70) | Reference | 1.43 (1.14-1.80) |
|  | Elevated FPG | 0.95 (0.64-1.40) | Reference | 1.42 (1.09-1.85) |
|  | Elevated TG | 0.88 (0.61-1.27) | Reference | 0.84 (0.64-1.10) |
|  | Reduced HDL | 0.78 (0.62-0.98) | Reference | 0.94 (0.79-1.11) |

MetS: metabolic syndrome; BP: blood pressure; FPG: fasting plasma glucose; TG: triglycerides; HDL: high-density lipoprotein
