# Supplementary material for: Association between self-reported eating speed and metabolic syndrome in a Beijing adult population: a cross-sectional study
Source: BMC Public Health. 2018 Jul 11;18:855. doi: 10.1186/s12889-018-5784-z (PMC6042428; doi:10.1186/s12889-018-5784-z)
Supplement: Supplementary file 3 — Table S3. The adjusted association between MetS, obesity and eating speed. (DOCX 14 kb) [file 12889_2018_5784_MOESM3_ESM.docx]

**Table S3** The adjusted association between MetS, obesity and eating speed

| Gender | Variable | Eating speed | | | *P* for trend |
| --- | --- | --- | --- | --- | --- |
|  |  | Slow | Medium | Fast |  |
| All | MetS | Reference | 1.63 (1.25-2.12) | 2.08 (1.59-2.72) | <0.0001 |
|  | Obesity | Reference | 1.25 (0.86-1.82) | 1.87 (1.29-2.72) | <0.0001 |
| Male | MetS | Reference | 1.51 (1.10-2.06) | 1.94 (1.42-2.67) | <0.0001 |
|  | Obesity | Reference | 1.12 (0.73-1.71) | 1.64 (1.08-2.50) | 0.0003 |
| Female | MetS | Reference | 1.82 (1.11-2.98) | 2.22 (1.33-3.73) | 0.0034 |
|  | Obesity | Reference | 1.72 (0.77-3.84) | 2.77 (1.22-6.27) | 0.0042 |

MetS and obesity were diagnosed using BMI equal or above 30 kg/m^2^ as the criteria

MetS: metabolic syndrome; BMI: body mass index
